# Decoding electromechanical coupling in shaker potassium ion channel: Intermediate voltage sensor and close pore

**DOI:** 10.1063/4.0001151

**Published:** 2025-10-27

**Authors:** Richa Agrawal, Ramon Mendoza Uriarte, Bernardo Pinto, Trayder Thomas, Francisco Bezanilla, Eduardo Perozo, Benoit Roux

**Affiliations:** Department of Biochemistry and Molecular biology, University of Chicago,, Chicago, IL, USA

## Abstract

Voltage-gated potassium (Kv) channels are essential for neuronal excitability, with activation involving tightly coupled movements of the voltage- sensing domain (VSD) and pore domain (PD). The Shaker ILT mutant (V369I, I372L, S376T) has been extensively used to dissect this coupling, as it exhibits a striking decoupling of voltage sensor movement from pore opening—approximately 80% of VSD activation is functionally uncoupled from pore gating, while the remaining 20% represents a rate-limiting, coupled transition. Although functionally intriguing, the structural basis of this partial electromechanical coupling has remained elusive. In this study, we determined the structure of the Shaker ILT mutant using single-particle cryo-electron microscopy (cryo-EM) in detergent micelles at a resolution of 3.4 Å. Structural comparison with the wild-type Shaker channel revealed a pronounced tilt of the S4 helix within the VSD and ∼5 Å downward displacement of the entire VSD, accompanied by a closed conformation of the S6 activation gate. These structural changes correlate with the functionally decoupled state. Additionally, restrained Alpha Fold and MD simulations combine approach were used on ILT structure to observe further VSD downward shift. Together, our results provide structural evidence supporting the decoupling of voltage sensor movement from pore opening in the ILT mutant and reveal key conformational features underlying this unique gating behavior. These findings advance our understanding of electromechanical coupling mechanisms in Kv channels.

